# Supplementary material for: Epidemiological modelling of the 2005 French riots: a spreading wave and the role of contagion
Source: Sci Rep. 2018 Jan 8;8:107. doi: 10.1038/s41598-017-18093-4 (PMC5758762; doi:10.1038/s41598-017-18093-4)
Supplement: Supplementary file 5 — Supplementary Information [file 41598_2017_18093_MOESM5_ESM.pdf]

Epidemiological modelling of the 2005 French riots: a spreading wave and the role of contagion

Laurent Bonnasse-Gahot, Henri Berestycki, Marie-Aude Depuiset, Mirta B. Gordon, Sebastian Roché, Nancy Rodriguez, Jean-Pierre Nadal

Supplementary Information

Supplementary Figures

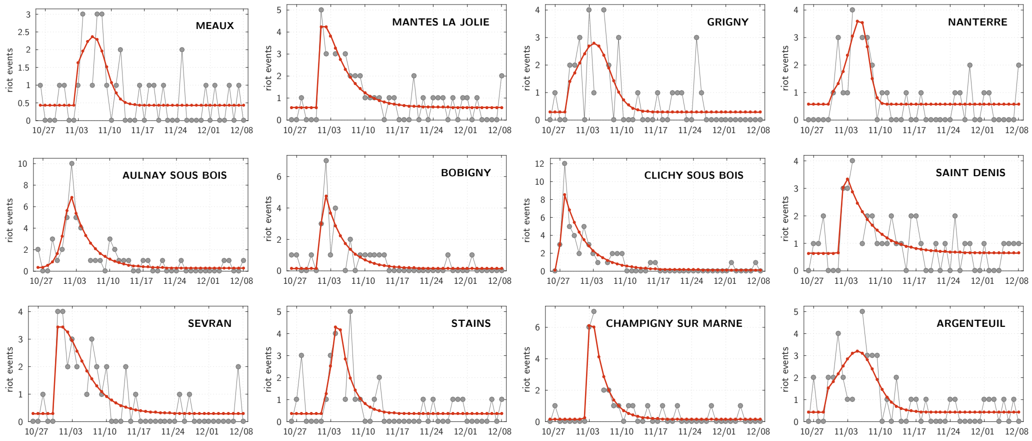

Figure S1: The 2005 French riots: data and single site fits for the 12 most active Île-de-France municipalities. Dots: number of events. Continuous curve: fit with the single site SIR model.

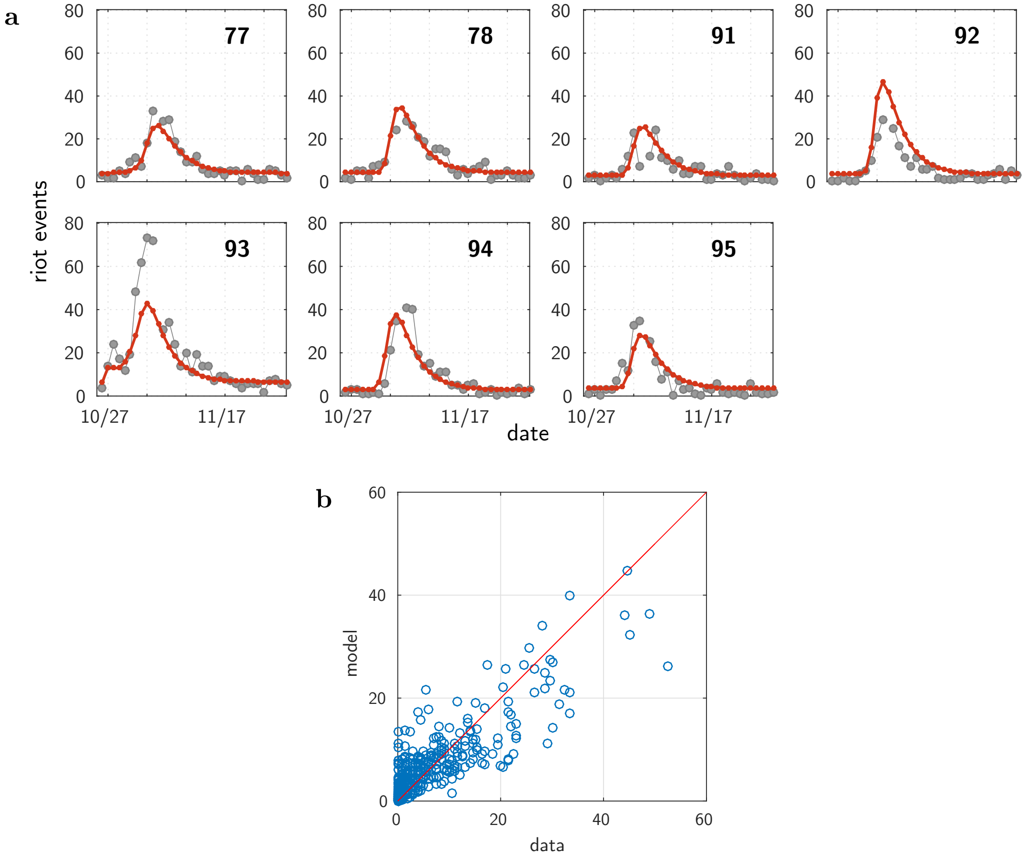

Figure S2: Control numerical experiment: fit with an inadequate reference population. Here the reference population is taken as the total population. (a) Île-de-France municipalities (aggregated by départements): data (dots), model (continuous curve) making use of the inadequate reference population. (b) Total number of events, model vs. data. Each dot represents one municipality. These results should be compared with the one on Fig. 2 of the main text – apart from the choice of a different reference population, the model options (choice of Ψ and of the weights) are the same.

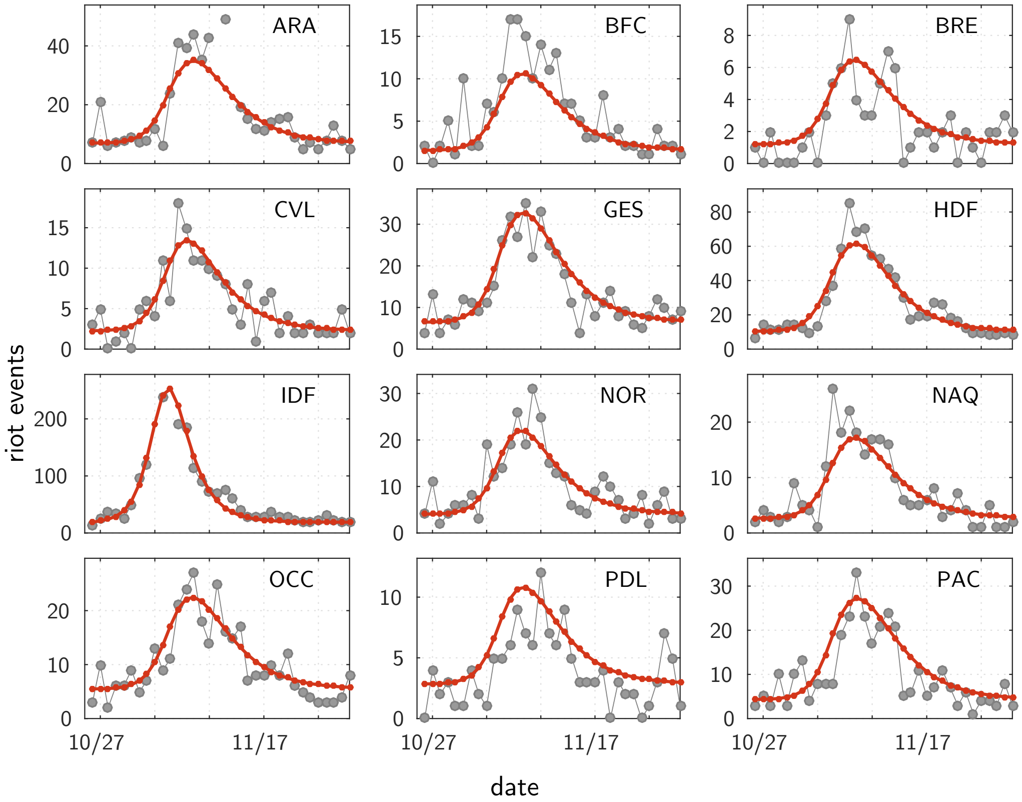

Figure S3: Results: all of France, spatial SIR model, calibration at the scale of the départements. Results aggregated by 'régions' (as of 2016), taking into account all the départements. See main text, section *Fitting the data: the wave across the whole country*. See Supplementary Table S2 for more details on the régions.

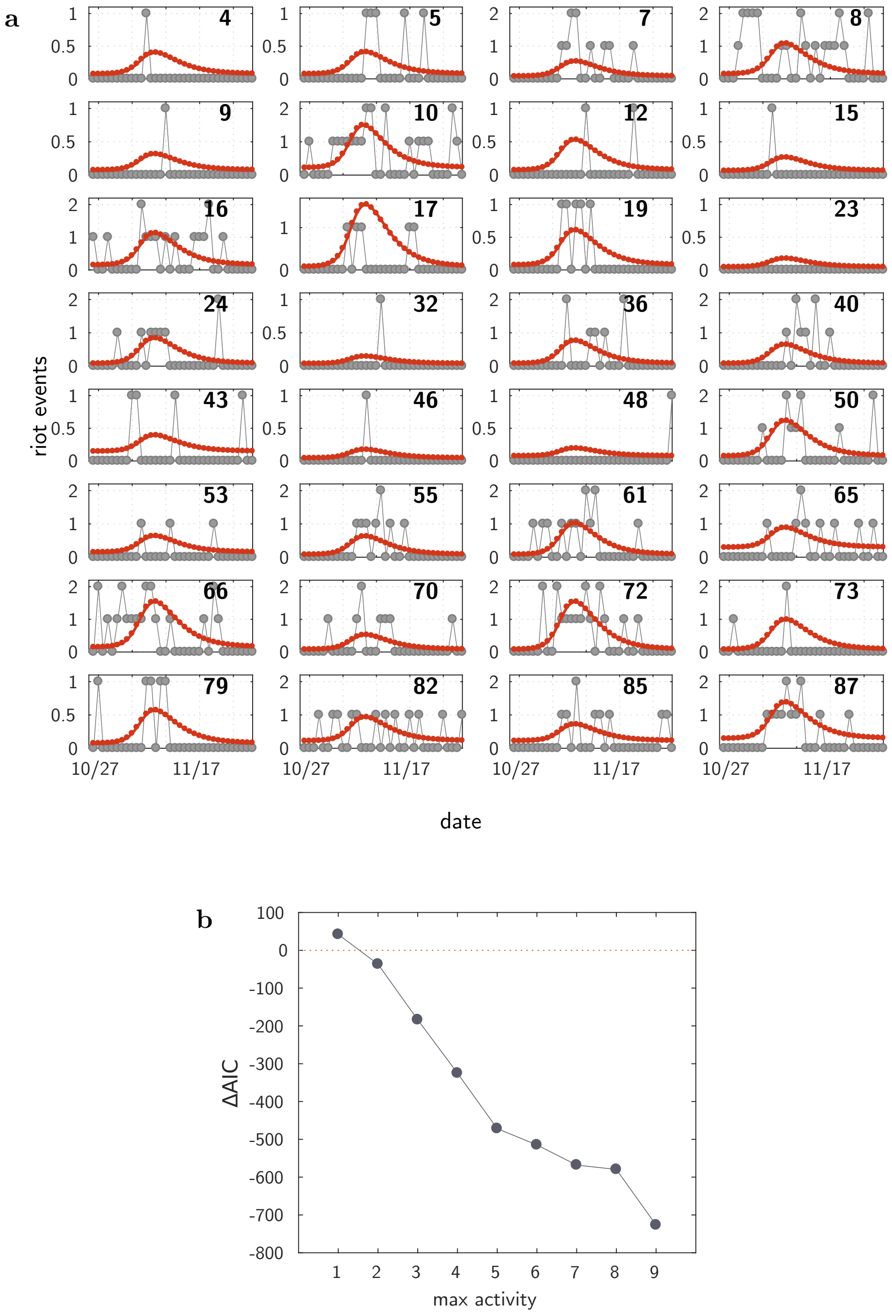

Figure S4: Results, calibration at the scale of the départements: minor sites. Even where the number of events is very small, the model predicts the sites to be hit by the wave, with a small amplitude and at the correct period of time. (a) All the sites where the number of events on any given day is inferior or equal to 2. (b) Comparison between our model and a constant rate null-hypothesis model. The y-axis corresponds to the difference in Akaike Information Criterion. A negative value of ΔAIC means a better performance of our contagion model. The x-axis indicates the maximum activity considered (*ie* all the sites with maximum daily activity inferior or equal to the corresponding value are used in the computation of the difference in AIC). For more details, see Main text, Materials and Methods, section *Minor sites: Comparison with a constant rate null-hypothesis*.

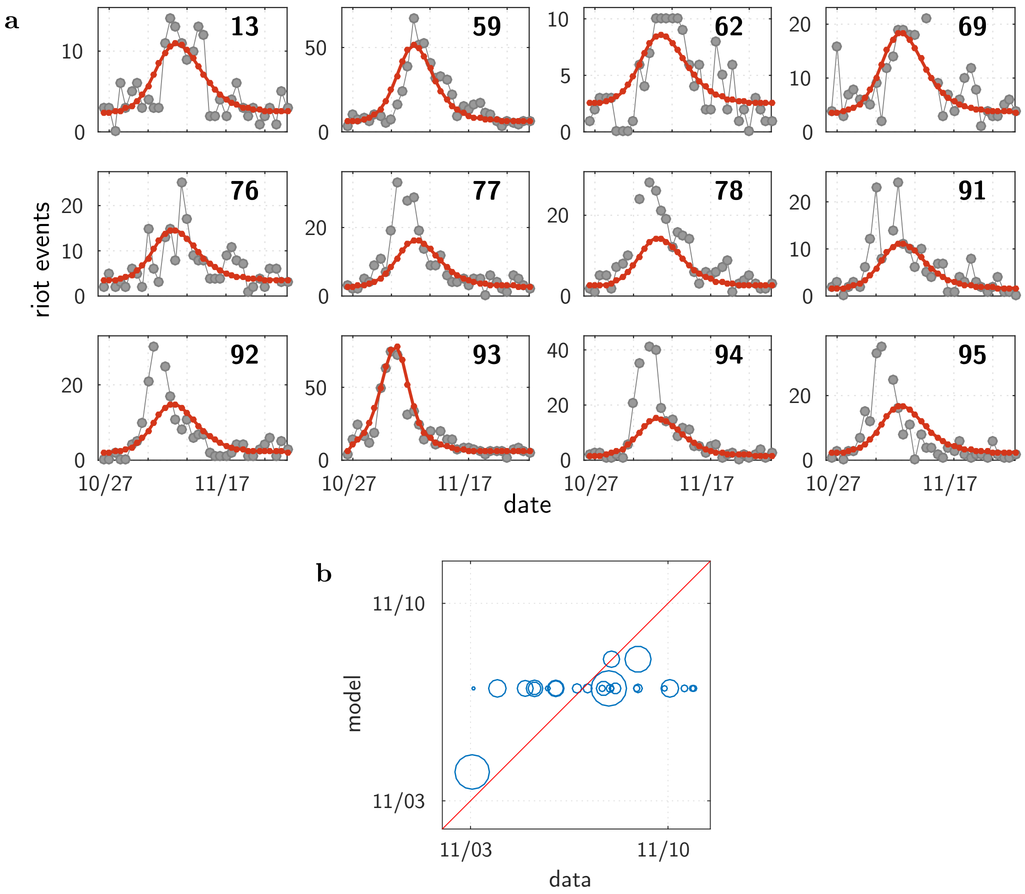

Figure S5: Distance-independent null hypothesis model. This figure, to be compared with Fig. 4, main text, shows that the absence of geographic dependency in the contagion process fails to reproduce the wave, but also to account for the amplitudes of the riots (see Materials and Methods for details). Plotted here: All of France, model calibration at the scale of the départements. (a) Time course of the riots in France: data (dots) and model (continuous curves). Only the 12 most active départements are shown. (b) Temporal unfolding (date when the number of riot events reaches its maximum value), shown for the départements having more than 60 events. Each blue circle has a diameter proportional to the reference population of the corresponding départements. The red lines depict the identity diagonal line.

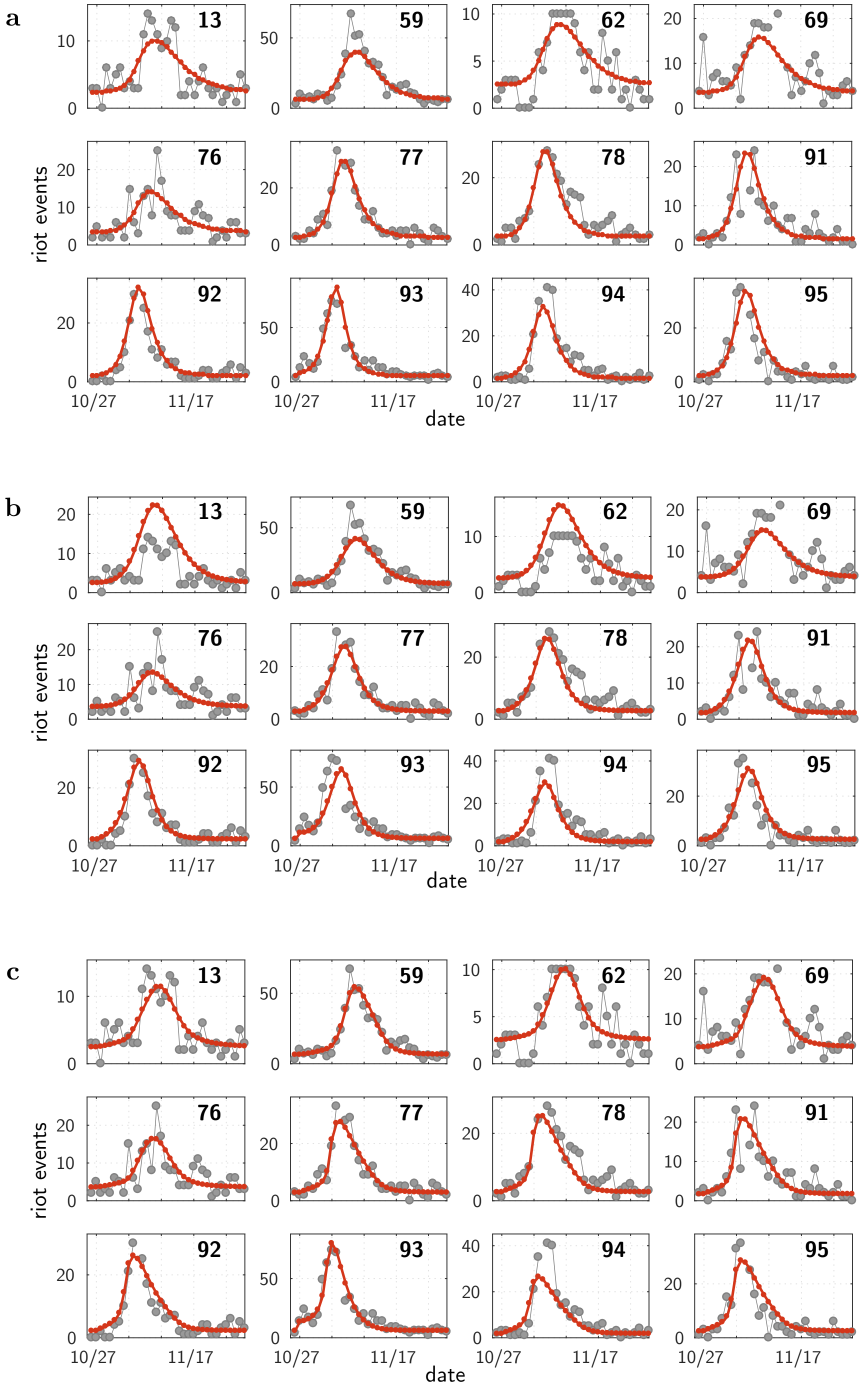

Figure S6: Model variants. All of France, model calibration at the scale of the départements. (a) For ease of comparison, reproduction of Fig. 4b (model with 9 free parameters); (b) and (c), same model as (a), except: (b) no extra specific susceptibility value β (6 free parameters), (c) the use of a sigmoidal function for Ψ (12 free parameters).

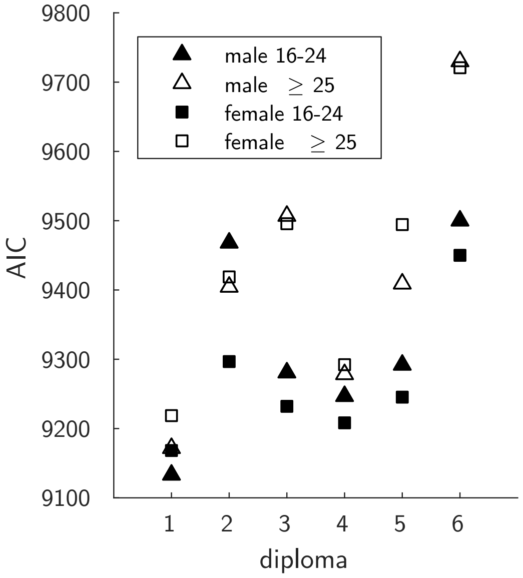

Figure S7: Choice of the reference population. Model comparison, considering cross-linked database that involve age, sex and diploma (INSEE statistics of 2006). All of France, model calibration at the scale of the départements. For each point, the model is the minimal one, using the 6 free parameters ω, A, ζ_0, d_0, ξ, β, and the corresponding reference population. For the Akaike Information Criterion (AIC), the lower is the better. Diploma categories: 1, no diploma; from 2 to 6, higher and higher levels of education, with 5, high school diploma, 6 university diploma (specific French levels: 2: CEP; 3: BEPC; 4: CAP-BEP; 5: Bac général, bac technique; 6: Diplôme universitaire 1er, 2ème ou 3ème cycle, BTS-DUT).

Supplementary Tables

Description of the model	Number of free parameters	List of parameters	AIC
model presented in the paper (see Fig. 2)<br>Ψ strict threshold, weights W_{kj} power law	8	ω, A, ζ_0, d_0, δ, η, γ, Λ_c	9718
same, but with Ψ linear	6	ω, A, ζ_0, d_0, δ, β	9789
same, but with weights W_{kj} exponential decay + constant	8	ω, A, ζ_0, d_0, ξ, η, γ, Λ_c	9738
same, but with total population as reference population (see Fig. S2)	8	ω, A, ζ_0, d_0, δ, η, γ, Λ_c	10055

(a) Model comparison. Île-de-France région, model calibration at the scale of municipalities.

Description of the model	Number of free parameters	List of parameters	AIC
model presented in the paper (see Fig. 4)<br>Ψ linear, weights W_{kj} exponential decay + constant<br>outliers defined with a three standard deviations criterion	9	ω, A, ζ_0, d_0, ξ, β, β_{13}, β_{62}, β_{93}	8979
same, but weights W_{kj} power law	9	ω, A, ζ_0, d_0, δ, β, β_{13}, β_{62}, β_{93}	9000
same, but weights W_{kj} self vs rest<br>(distance-independent null hypothesis model see Fig. S5)	8	ω, A, ζ_0, ξ, β, β_{13}, β_{62}, β_{93}	9419
same, but with densities instead of numbers	9	ω, A, ζ_0, d_0, ξ, β, β_{13}, β_{62}, β_{93}	9080
same, but no extra susceptibility value β (see Fig. S6b)	6	ω, A, ζ_0, d_0, ξ, β	9133
same, but with one susceptibility value β<br>outliers defined with a four standard deviations criterion	7	ω, A, ζ_0, d_0, ξ, β, β_{13}	9058
same, but with Ψ sigmoidal<br>(see Fig. S6c)	12	ω, A, ζ_0, d_0, ξ, β, τ, γ, Λ_c, β_{13}, β_{62}, β_{93}	8923

(b) Model comparison. All of France, model calibration at the scale of the départements.

Table S1: Summary of miscellaneous model variants that were tested. In both sub-tables, "same" means same model as the one presented in the main text and described in the first row, except for the described option. See Material and Methods for more details. For convenience, we give below a brief reminder of the equations of some options for the interaction weights (W_{kj} in $\Lambda_k(t) = \sum_j W_{kj}\lambda_j(t)$), and for the function Ψ used to model the probability for a susceptible agent to become a rioter.

Function Ψ:

- linear: $\Psi(\Lambda) = \beta\Lambda$
- strict threshold: $\Psi(\Lambda) = 0$ if $\Lambda \leq \Lambda_c$; $\Psi(\Lambda) = \eta\,(1 - \exp -\gamma\,(\Lambda - \Lambda_c))$ if $\Lambda > \Lambda_c$
- sigmoidal: $\Psi(\Lambda) = \beta(1 - \exp -\tau\Lambda)\,(1 - \exp -\gamma\,(\Lambda - \Lambda_c))^{-1}$

Weights:

- power law: $W_{kj} = (1 + \text{dist}(k,j)/d_0)^{-\delta}$
- exponential decay + constant: $W_{kj} = \xi\; +\; (1-\xi)\exp\left(-\text{dist}(k,j)/d_0\right)$
- self vs rest: $W_{kj} = 1$ if $j = k$; $W_{kj} = \xi$ otherwise.

code	full name	départements
ARA	Auvergne-Rhône-Alpes	01 03 07 15 26 38 42 43 63 69 73 74
BFC	Bourgogne-Franche-Comté	21 25 39 58 70 71 89 90
BRE	Bretagne	22 29 35 56
CVL	Centre-Val de Loire	18 28 36 37 41 45
GES	Grand Est	08 10 51 52 54 55 57 67 68 88
HDF	Hauts-de-France	02 59 60 62 80
IDF	Île-de-France	75 77 78 91 92 93 94 95
NOR	Normandie	14 27 50 61 76
NAQ	Nouvelle-Aquitaine	16 17 19 23 24 33 40 47 64 79 86 87
OCC	Occitanie	09 11 12 30 31 32 34 46 48 65 66 81 82
PDL	Pays de la Loire	44 49 53 72 85
PAC	Provence-Alpes-Côte d'Azur	04 05 06 13 83 84

Table S2: Code ISO 3166-2 (without the prefix FR-) and associated name of the 12 régions of Metropolitan France (as of 2016, excluding Corsica), along with the ID number of the départements that they encompass. For the ISO codes, see `https://www.iso.org/obp/ui/#iso:code:3166:FR`.

Supplementary Videos

• Supplementary Video 1. **Riot propagation around Paris: smoothed data.**
This video shows the riot propagation around Paris. The map shows the municipality boundaries, with Paris at the centre. For each municipality for which data is available, a circle is drawn with an area proportional to the estimate of the size of the susceptible population (see main text, section Methods). Instead of making use of the raw data, for each municipality we replaced each day value by the one given by the fit with the single site epidemic model considered here as a tool for smoothing the data. The colour represents the intensity of the rioting activity: the warmer the colour, the higher the activity. The pace of the video corresponds to three days per second. In order to improve the fluidity of the video, we increased the number of frames per second by interpolating each day with 7 new frames, whose values are computed thanks to a piece-wise cubic interpolation of the original ones. The resulting frame rate is then 24 frames per second.
The maps have been generated with the Mapping toolbox of the MATLAB software making use of the Open Street Map data ©OpenStreetMap contributors (`https://www.openstreetmap.org/copyright`).
The video is encoded with the open standard H.264.

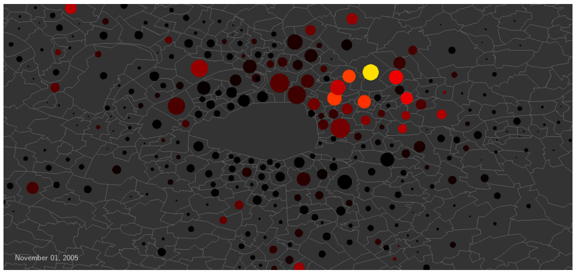

Still image from SI Video 1

• Supplementary Video 2. **Riot propagation around Paris: model with non-local contagion.**
This video shows the riot activity as predicted by the data-driven global epidemic-like model. Same technical details as for the SI Video 1.
The maps have been generated with the Mapping toolbox of the MATLAB software making use of the Open Street Map data ©OpenStreetMap contributors (`https://www.openstreetmap.org/copyright`).
The video is encoded with the open standard H.264.

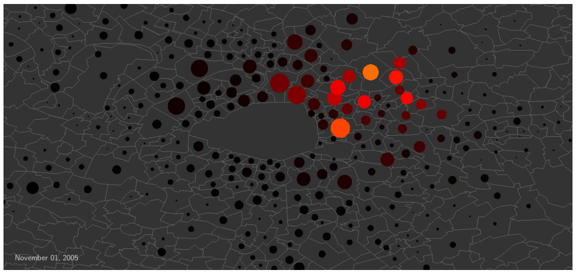

Still image from SI Video 2

• Supplementary Video 3. **Spatial SIR: wave propagation in a homogeneous medium.**
We illustrate the formal continuous spatial SIR model with a video showing the propagation of a wave. The underlying medium is characterized by a uniform density of susceptible individuals. The weights $w(x-y)$ in the interaction term are given by a decreasing exponential function of the Euclidean distance $||x-y||$.
The video is encoded with the open standard H.264.

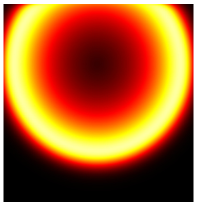
Still image from SI Video 3

• Supplementary Video 4. **Spatial SIR: wave propagation in a non-homogeneous medium.**
Same as SI Video 3, but with a heterogeneous density of susceptible individuals, characterized by (1) a decrease of the density towards 0 near the boundary of the image, so that the wave dies before leaving the frame, (2) a hole at the centre, which is then bypassed by the wave, (3) a concentration of susceptible individuals that globally decreases on the y-axis, so that the wave dies while going downward.
The video is encoded with the open standard H.264.

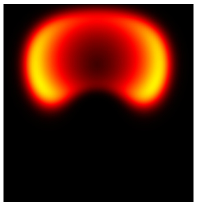
Still image from SI Video 4
